# Supplementary material for: Comparative multiomics analysis of cell physiological state after culture in a basket bioreactor
Source: Sci Rep. 2022 Nov 23;12:20161. doi: 10.1038/s41598-022-24687-4 (PMC9686226; doi:10.1038/s41598-022-24687-4)
Supplement: Supplementary file 1 — Supplementary Information 1. [file 41598_2022_24687_MOESM1_ESM.zip › raw data/Transcriptomic raw data/7.AS/2.dasplot/TE1vsTC1/RI/16_CAPRIN2_NW_023666069.1_4554765_4554965_+@NW_023666069.1_4554765_4556367_+@NW_023666069.1_4556152_4556367_+.pdf]

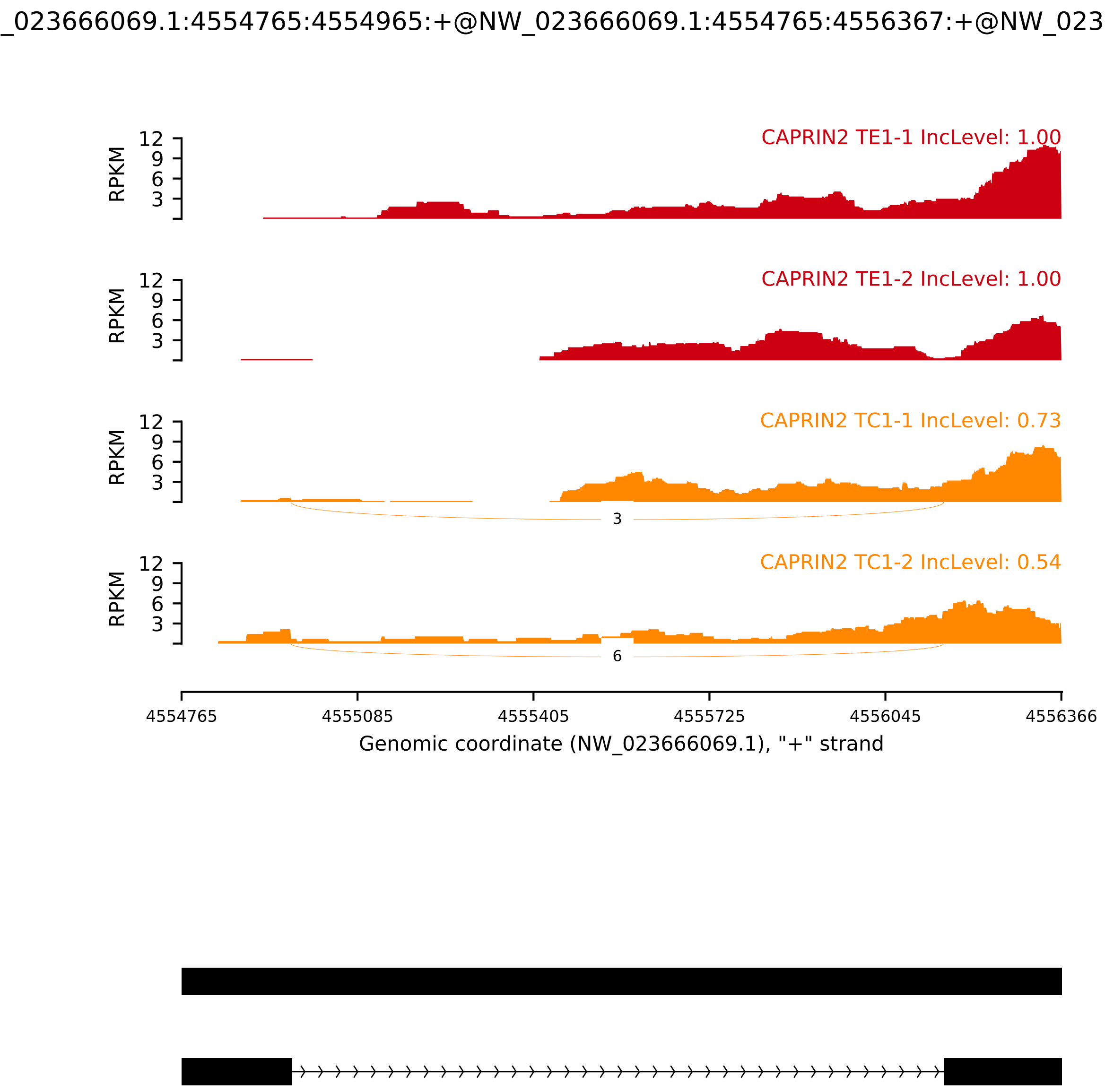
_023666069.1:4554765:4554965:+@NW_023666069.1:4554765:4556367:+@NW_023666069.1:4556152:455636
CAPRIN2 TE1-1 IncLevel: 1.00
CAPRIN2 TE1-2 IncLevel: 1.00
CAPRIN2 TC1-1 IncLevel: 0.73
CAPRIN2 TC1-2 IncLevel: 0.54
RPKM
12
9
6
3
3
6
4554765
4555085
4555405
4555725
4556045
4556366
Genomic coordinate (NW_023666069.1), "+" strand
